# Decreased Toll-like receptor 8 expression and lower TNF-alpha synthesis in infants with acute RSV infection

**DOI:** 10.1186/1465-9921-11-143

**Published:** 2010-10-14

**Authors:** Kreso Bendelja, Valerija Vojvoda, Neda Aberle, Jasna Cepin-Bogovic, Alenka Gagro, Gordana Mlinaric-Galinovic, Sabina Rabatic

**Affiliations:** 1 Department of Cellular Immunology, Institute of Immunology, Zagreb, Croatia; 2 Department of Pediatrics, General Hospital "Dr. Josip Benčević", Slavonski Brod, Croatia; 3 Department of Pulmonology, Allergology, Immunology and Rheumatology, University Children's Hospital, Zagreb, Croatia; 4 Department of Virology, Croatian National Institute of Public Health, Zagreb, Croatia

## Abstract

**Background:**

Toll-like receptors (TLRs) are part of the innate immune system, able to recognize pathogen-associated molecular patterns and activate immune system upon pathogen challenge. Respiratory syncytial virus (RSV) is a RNA virus particularly detrimental in infancy. It could cause severe lower respiratory tract disease and recurrent infections related to inadequate development of anti-viral immunity. The reason could be inadequate multiple TLRs engagement, including TLR8 in recognition of single-stranded viral RNA and diminished synthesis of inflammatory mediators due to a lower expression.

**Methods:**

Intracellular TLR8 expression in peripheral blood monocytes from RSV-infected infants was profiled and compared to healthy adults and age matched controls. Whether the observed difference in TLR8 expression is a transitory effect, infants in convalescent phase (4-6 weeks later) were retested. Specific TLR8-mediated TNF-α production in monocytes during an acute and convalescent phase was analyzed.

**Results:**

RSV-infected and healthy infants had lower percentage of TLR8-expressing monocytes than healthy adults whereas decreased of TLR8 protein levels were detected only for RSV-infected infant group. Lower protein levels of TLR8 in monocytes from RSV-infected infants, compared to healthy infants, negatively correlated with respiratory frequency and resulted in lower TNF-α synthesis upon a specific TLR8 stimulation. In the convalescent phase, levels of TLR8 increased, accompanied by increased TNF-α synthesis compared to acute infection.

**Conclusions:**

Lower TLR8 expression observed in monocytes, during an acute RSV infection, might have a dampening impact on early anti-viral cytokine production necessary to control RSV replication, and subsequently initiate an adaptive Th1 type immune response leading to severe disease in infected infants.

## Background

Respiratory syncytial virus (RSV), an enveloped ssRNA pneumovirus of the Paramyxoviridae family, is an important cause of lower respiratory tract (LTR) infection in a small percentage of infants, although virtually all infants become infected. Frequent reinfections implicate inadequate development of immunological memory perhaps due to the virus-mediated subversion of innate and/or adaptive immune mechanisms [1-3]. In recent years, more effort has been made to explore innate immune mechanisms involved in immunopathological processes observed in severe LRT disease during primary infection in infants [4-6].

Innate cellular defense can be triggered by a variety of mechanisms, including host recognition of pathogen-associated molecular patterns (PAMPs) through pattern recognition receptors. Structural RSV glycoproteins are recognized by surface TLR2 and TLR4 [7-9], while viral RNA engages cytoplasmic retinoic acid inducible gene I (RIG-I) in infected epithelial cells (primary RSV target) [10-12]. However, in immune cells with ability to endocytose viral particles and present antigens, such as dendritic cells (DC) and monocytes/macrophages, viral RNA could be sensed via endosomal TLR3, TLR7 and TLR8 [11,13-15].

The cellular recognition of a virus activates multiple signaling pathways, such as NF-κB transcription factor and the interferon-regulatory factors (IRFs) [16], with subsequent release of multiple and potent antiviral cytokines, among them tumor necrosis factor alpha (TNF-α) and type I interferons (IFN) [17,18]. Myeloid dendritic cells (mDC) and monocytes/macrophages, activated via endosomal or cytoplasmic RNA sensors, also release IL-12p70 necessary for the activation of NK cells and cytotoxic T-lymphocytes and IFN-γ production [19-21]. IFN-γ is required for driving the Th1 type response, as opposed to inappropriate Th2 response detected in RSV infection [22].

Functional TLR studies in healthy newborns, a population with a risk to develop LRT infection encountering RSV, have revealed a decreased synthesis of antiviral cytokines implying an inadequate TLR activation [23-25]. Moreover, cord blood mononuclear cells express lowered levels of myeloid differentiation factor 88 [26], a central signaling adaptor molecule for the majority of TLRs (except for TLR3), that has a significant role in preventing severe disease development in RSV-murine model [27]. It seems that the absence of endosomal TLR-stimulation is responsible for the development of low affinity anti-RSV antibodies, causing severe disease upon infection upon vaccination with formalin inactivated virus [28].

The aim of this study was to determine the expression of TLR8 in peripheral blood monocytes during an acute severe RSV LRT infection and compare with a healthy infant/adult controls and infants in the convalescent phase 4-6 weeks later. Ability of monocytes to produce TNF-α upon short-time stimulation with specific TLR7/8 ligand was measured in the acute and convalescent phase. Possible correlation between the severity of disease and TLR expression in RSV-infected infants was also analyzed.

## Methods

### Subjects

Study encountered 15 infants, 10 boys and 5 girls (aged 1-8.5 months; median age 2.5 months), admitted to the University Hospital for Infectious Diseases and Children's Hospital in Zagreb, Croatia (Table 1). The infants suffered from RSV-caused bronchiolitis (defined as wheezing, hypoxia with O_2 _saturation < 95% and normal chest radiographs) or pneumonia (defined as crackles on auscultation with wheezing, and confirmed with chest radiographs showing infiltrates). The samples of blood (with and without heparin) and nasopharyngeal secretions were simultaneously obtained within 7 days from the onset of acute illness and 4-6 weeks after first sampling (N = 10). Laboratory parameters such as the quantity of C-reactive protein (CRP), erythrocyte sedimentation rate (ESR) and leukocyte count were used to exclude possible non-viral infections [29]. None of the tested infants received glucocorticoid drugs.

**Table 1:** Patients and clinical findings.

Clinical findings	Controls (n = 10)	Bronchiolitis (n = 12)	Pneumonia (n = 3)	Total (n = 15)
Age (months)	**5** (1-13)	**2.5** (1-8.5)	**1** (1-5)	**2.5** (1-8.5)

Gestational age	**40** (39-40)	**40** (36-40)	**38** (32-40)	**40** (32-40)

Birth weight (kg)	**3.4** (3.2-4.0)	**3.4** (2.8-4.1)	**3.1** (2.4-3.4)	**3.4** (2.4-4.1)

Mode of delivery (normal/caesarian surgery)	**9/1**	**11/1**	**2/1**	**13/2**

Apgar	**10** (9-10)	**10** (9-10)	**10** (8-10)	**10** (8-10)

WBC count (x10^9^/L)	**8.2** (4.3-14.7)	**12.1** (4.6-18.7)	**11** (9.4-13.2)	**11.8** (4.6-18.7)

CRP (mg/L)	**-**	**4.8** (1.2-8.9)	**2.1** (1.4-11)	**4.7** (1.2-11)

ESR (mm/h)	**-**	**10** (5-30)	**9** (5-25)	**9** (5-30)

Wheezing (No. of cases)	**-**	**12**	**3**	**15**

Wheezing duration (days)	**-**	**4.5** (1-6)	**1** (1-3)	**3** (1-6)

Hospital stay (days)	**-**	**7.5** (4-10)	**9** (7-10)	**8** (4-10)

MOS (%)	**-**	**91.5** (88-98)	**90** (83-91)	**91** (83-98)

MRR (/min)	**-**	**55** (40-60)	**50** (45-55)	**55** (40-60)

Oxygen supplementation (No. of cases)	**-**	**6**	**2**	**8**

Oxygen supplementation (days)	**-**	**1.5** (1-7)	**7** (6-8)	**4** (1-8)

Pulmonary X-ray scan (No. of cases)				

Negative	**-**	**12**	**0**	**12**

Infiltrate	**-**	**0**	**3**	**3**

Infants with pneumonia (defined as crackles on auscultation and chest radiographs showing infiltrates) had also developed bronchiolitis (defined as wheezing with hypoxia and O_2 _saturation < 95%). Medians, range (in parentheses) and the number of patients (*n*) were determined. Minimal oxygen saturation (MOS) was measured by percutaneous oxymetry breathing ambient air.

WBC - white blood cells, CRP - C-reactive protein, ESR - erythrocyte sedimentation rate, MRR - maximal respiratory rate (frequency) during hospitalization period

Control blood samples were obtained from healthy infants and adults (N = 10, aged 21-39 years). A healthy infant group consisted of 10 infants (aged 1-13 months; median age 5 months) hospitalized for minor surgery, free from allergic, immune and hematological disorders.

Patients and controls were included in the study after written consent had been obtained from their parents/guardians or from themselves. The study was approved by the Medical Ethics Committees of both hospitals.

### Virus identification

RSV infection was confirmed by detecting the virus in nasopharyngeal secretion by direct immunofluorescence test (Light diagnostics, Temecula, USA).

### Isolation of PBMC and cryopreservation

For the analysis of TLR8 expression, cryopreserved peripheral blood mononuclear cells (PBMC) obtained from fresh heparinized blood upon density gradient isolation were used. Briefly, blood was diluted in PBS to 1:1 volume ratio and centrifuged for 30 minutes at 800 g on Ficoll-Paque Plus (GE Healthcare Bio-Sciences AB, Uppsala, Sweden). Isolated PBMC were resuspended in ice-cold freezing media (10% RPMI 1640, 80% fetal bovine serum and 10% dimethyl sulphoxide) at 2 × 10^6 ^cells/mL concentration. Aliquots of cell suspension (1.0 mL) were transferred to Nalgene Cryo freezing containers (Nalge Nunc International, Rochester, NY) and placed overnight in an -80°C freezer. The frozen specimens were transferred to liquid nitrogen within 24 h and maintained until thawed. In parallel, serum samples were obtained and kept frozen at -20°C.

### Intracellular TLR8 detection

The frozen PBMC were thawed in 37°C water bath with continuous agitation until completely melted and resuspended slowly in warm RPMI 1640 media supplemented with 10% FBS. The cells were centrifuged at 400 g for 5 minutes and then washed again in staining buffer (1% fetal calf serum, 0.1% NaN_3 _and Dulbecco's PBS) to completely remove traces of DMSO. Viability was estimated by trypan blue dye exclusion.

PBMC aliquots were placed in sterile 12 × 75-mm polystyrene round-bottomed tubes (Falcon, Becton Dickinson, Lincoln Park, USA) and incubated for 15 min in the dark at room temperature with antibodies for an anti-CD14 APC (Becton Dickinson, Heidelberg, Germany). The cells were washed with staining buffer, fixed with fixation buffer (4% formaldehyde in Dulbecco's PBS) for 30 min at 4°C, and washed with staining buffer. The cells were then washed with permeabilization buffer (1% FCS, 0.1% NaN_3 _and 0.1% saponin in Dulbecco's PBS) and stained with PE-conjugated anti-human TLR8 PE (Imgenex, San Diego, USA) for 30 min at 4°C. Unbound antibody was washed away by centrifugation in permeabilization buffer and cells were resuspended in staining buffer before analysis by flow cytometer. Isotype-matched controls for the surface and intracellular staining were included.

### PBMC culture and intracellular TNF-α production

The samples of fresh PBMC were resuspended in RPMI 1640 medium containing 10% autologus sera. Cell suspensions were incubated in a cultivating medium alone or with the addition of specific TLR-ligand. For TLR4 stimulation (positive control), 0.1 μg/mL lipopolysaccharide *E. coli *0111:B4 (LPS; Sigma, St Louis, USA) was dissolved in the cultivating medium, whereas TLR8 stimulation was achieved using 5 μg/mL thiazoloquinolone derivate (CL075; Invivogen, San Diego, USA), respectively, for 6 h at 37°C in 5% CO_2 _atmosphere, in the presence of 10 μg/mL brefeldin A (BFA; Sigma). Additionally, 100 μM chloroquine (Sigma) was added to designated tubes one hour before the activation to block TLR signaling and subsequent cytokine production.

After stimulation, the cells were fixed in fixation buffer for 30 minutes and stored at -80°C until further processed as follows: frozen fixed cells were thawed in 37°C water bath with continuous agitation until completely melted and resuspended slowly in staining buffer. The cells were centrifuged at 400 g for 5 minutes. After washed in permeabilization buffer, cells were labeled intracellulary with APC-conjugated anti-TNF-α (Becton Dickinson, Heidelberg, Germany) and FITC-conjugated anti-CD68 (AbD Serotec, Oxford, UK) monoclonal antibodies for 30 minutes at 4°C. Myeloid maker CD68 over CD14 staining was chosen since a robust monocyte stimulation causes CD14 downregulation (Additional file 1: Fig. S1 and Additional file 2: Fig. S2). Unbound antibody was washed away by centrifugation in permeabilization buffer and the cells were resuspended in staining buffer before analysis by flow cytometer. APC- and FITC- conjugated isotype-matched control antibodies were included.

### Flow cytometry

Cell samples were analyzed using CellQuest Software on a FACSCalibur flow cytometer (Becton Dickinson, Mountain View, USA). The forward and right-angle scatters were used to establish leukocyte subpopulation gates. The analysis included collecting 2 000 events in monocyte gate. Monocytes were designated as CD14^+ ^cells for the TLR8 expression analysis whereas CD68^+ ^cells were acquired for TNF-α analysis. TLR mean fluorescence intensity (MFI) values were calculated as difference between TLR-specific and unspecific isotype control antibody MFIs [30].

### Statistical analysis

The non-parametric Kruskal-Wallis ANOVA and Mann-Whitney U-test were used inter-group statistical analysis. *p*-values were corrected for multiple inter-group comparisons. Statistical relationship between variables was assessed by means of the Spearman rank order coefficient. Statistical tests were part of the software package Statistica v6.0 (Statsoft Inc., Tulsa, USA).

## Results

### Anti-TLR8 antibodies recognize functional receptors

**Figure 1:**
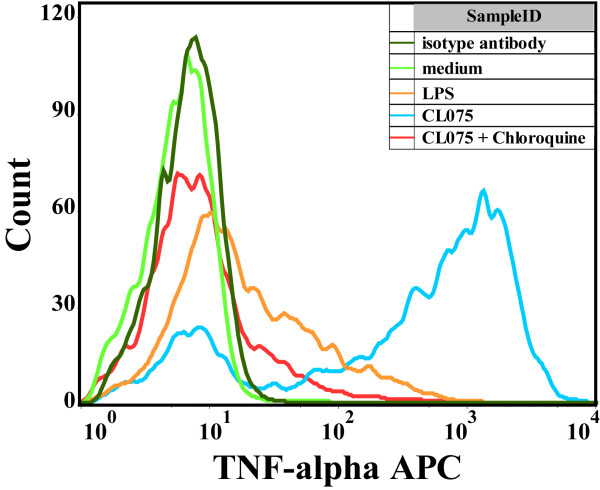
**Representative expression of TLR8 in monocytes**. Monocytes from healthy adults were stained with anti-CD14 APC-conjugated antibody and intracellular with anti-TLR8 PE-conjugated monoclonal antibody.

**Figure 2:**
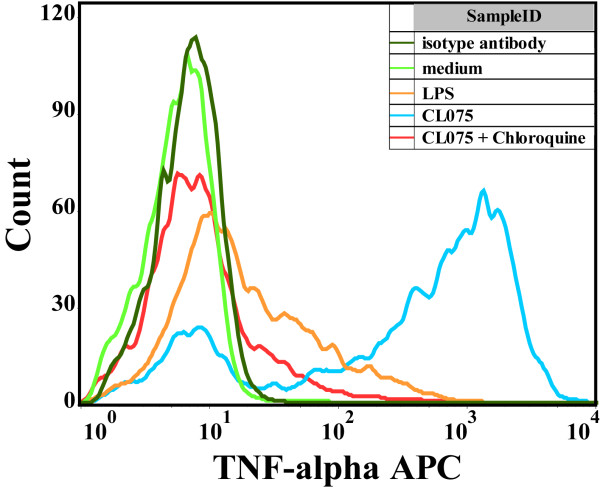
**Functional ability of monocytes to respond to specific TLR8 stimulation and produce TNF-alpha**. The monocytes were stimulated with 5 μg/mL CL075 and 100 ng/mL LPS for 6 h. Brefeldin A was added to inhibit cytokine release in the supernatant and intracellular TNF-α was stained with anti-TNF-α APC-conjugated antibody in CD68-positive monocytes. In separate experiments PBMC were pretreated for 1 hour with chloroquine prior CL075 stimulation.

The protein expression of TLR8 in peripheral blood monocytes was assessed by flow cytometer. TLR8 protein was expressed intracellulary (Figure 1) in the majority of circulating monocytes, whereas cell surface expression was not observed in *ex vivo *samples (data not shown). Functional ability of TLR8 to trigger TNF-α synthesis upon specific ligand binding was tested by CL075 stimulation of fresh PBMCs. Monocytes rapidly responded to LPS stimulation (positive control), as well as to CL075 (Figure 2) stimulation. The used concentrations of TLR-ligands represent optimal doses for specific stimulation of monocytes as extrapolated from optimization experiments (Additional file 3: Fig. S3). To determine TLR-independent activation by CL075 ligands, chloroquine pretreatment was introduced to inhibit endosomal pH-depended TLR8 signaling. Chloroquine addition completely abrogated TLR8-mediated TNF-α production (Figure 2).

### Decreased expression of TLR8 in monocytes from RSV-infected infants

**Figure 3:**
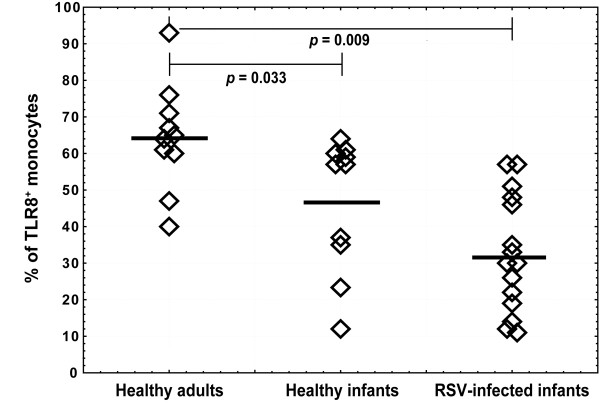
**Frequencies of monocytes expressing TLR8 among healthy adults, healthy infants and RSV-infected infants**. Isolated PBMC from healthy adults (n = 10), age-matched healthy infants (n = 10) and infants with acute RSV infection (n = 15) were surface stained for CD14 and intracellular for TLR8. Non-parametric Kruskal-Wallis ANOVA test was acquired to confirm statistical significance and Mann-Whitney U-test was applied for intergroup analysis. Bold lines represent median values. Whiskers indicate statistically significant difference between marked groups.

The study involved intracellular TLR8 staining coupled with surface marker analysis for CD14^+ ^monocytes in PBMC from RSV-infected infants, healthy infants and healthy adults. In comparison to healthy adults, healthy infants had slightly lower percentages of peripheral blood monocytes that expressed TLR8, while in RSV-infected infant group percentages of TLR8-positive monocytes were the lowest (Figure 3).

**Figure 4:**
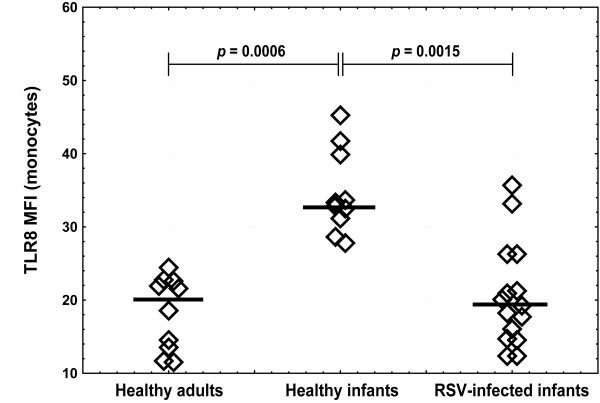
**TLR8 expression levels in monocytes**. Isolated PBMC from healthy adults (n = 10), age-matched healthy infants (n = 10) and infants with acute RSV infection (n = 15) were surface stained for CD14 and intracellular for TLR8. CD14-positive monocytes were analyzed for TLR8 level (mean fluorescence intensity). Non-parametric Kruskal-Wallis ANOVA test was acquired to confirm statistical significance and Mann-Whitney U-test was applied for intergroup analysis. Bold lines represent median values. Whiskers indicate statistically significant difference between marked groups.

A possible difference in TLR8 expression was also assessed by analyzing MFI values (which corresponds to TLR8 protein expression level per cell) for RSV-infected infants, healthy infants and adults. TLR8 expression levels in monocytes were significantly lower in RSV-infected than in healthy infants and healthy adults (Figure 4). Among all subject groups, monocytes from healthy infants had the highest TLR8 expression levels.

### Lower TLR8-mediated TNF-α synthesis in monocytes from RSV-infected infants

**Figure 5:**
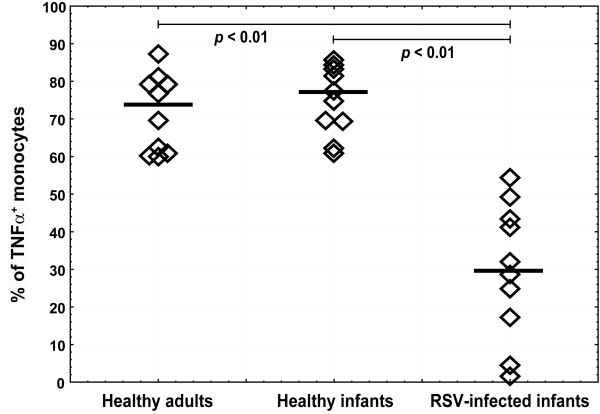
**Decreased percentages of monocytes producing TNF-alpha in RSV-infected infants**. Fresh PBMC from healthy adults (n = 10), age-matched healthy infants (n = 10) and infants with acute RSV infection (n = 10) were stimulated with 5 μg/mL CL075 for 6 h in the presence of brefeldin A. Intracellular TNF-α was stained with anti-TNF-α APC-conjugated antibody in CD68-positive monocytes upon permeabilization. Non-parametric Kruskal-Wallis ANOVA test was acquired to confirm statistical significance and Mann-Whitney U-test was applied for intergroup analysis.

**Figure 6:**
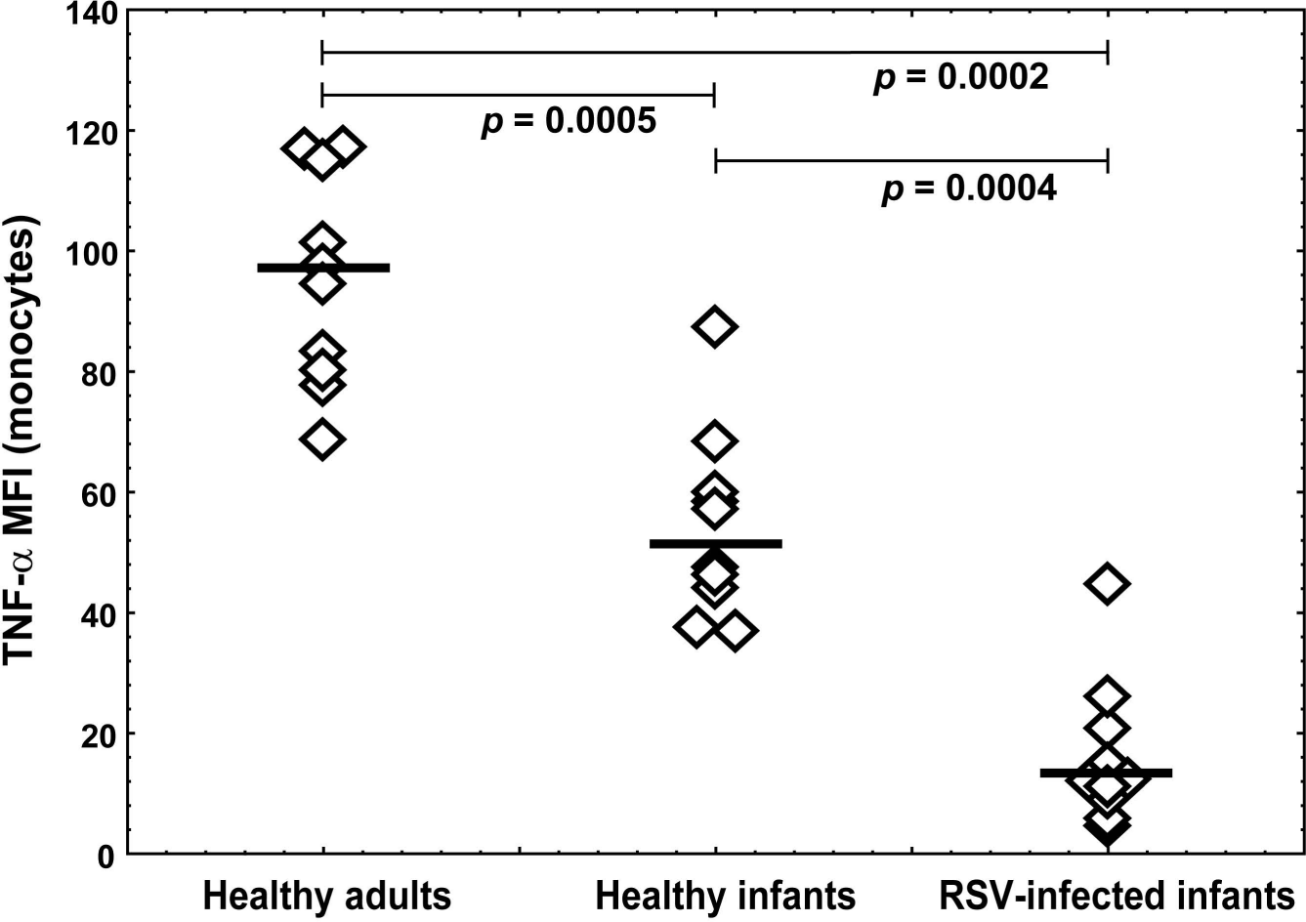
**Decreased TNF-alpha levels in monocytes from RSV-infected infants**. Fresh PBMC from healthy adults (n = 10), age-matched healthy infants (n = 10) and infants with acute RSV infection (n = 10) were stimulated with 5 μg/mL CL075 for 6 h in the presence of brefeldin A. Intracellular TNF-α was stained with anti-TNF-α APC-conjugated antibody in CD68-positive monocytes upon permeabilization. Non-parametric Kruskal-Wallis ANOVA test was acquired to confirm statistical significance and Mann-Whitney U-test was applied for intergroup analysis.

Functional ability of TLR8 expressed in monocytes, to trigger TNF-α synthesis upon specific TLR8 ligand binding was tested by CL075 stimulation of fresh PBMC samples from infants in acute RSV infection, healthy infants and adults. Monocytes rapidly responded to CL075 and accumulated TNF-α during 6 h of culture. Percentages of monocytes synthesized TNF-α were significantly lower in RSV-infected infant group than in healthy infants and adults (Figure 5). However, TNF-α expression levels in healthy adults were higher than in healthy infants (MFI median 96.2 vs. 52.3) that further decreased in RSV-infected infants (MFI median 12.2) (Figure 6).

### Higher TLR8 expression and TNF-α synthesis in the convalescent phase

**Figure 7:**
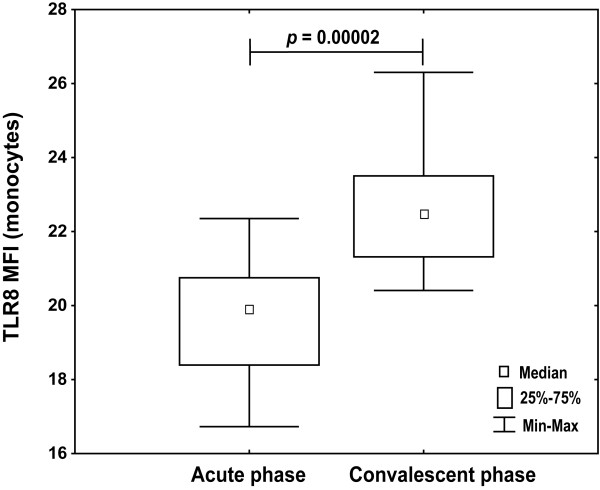
**Higher TLR8 expression levels in the convalescent phase**. Isolated PBMC from infants with acute RSV infection (n = 10) and in convalescent phase 4-6 weeks after first sampling were surface stained for CD14 and intracellular for TLR8. CD14-positive monocytes were analyzed for TLR8 expression level (mean fluorescence intensity). Non-parametric Kruskal-Wallis ANOVA test was acquired to confirm statistical significance and Mann-Whitney U-test was applied for intergroup analysis.

TLR8 expression levels in monocytes from 10 retested infants in the convalescent phase (4-6 weeks after first sampling) were significantly higher than from infants with bronchiolitis (up to 7 days of disease onset) during acute RSV infection (Figure 7). Although TLR8 median expression level was higher in convalescent phase, it still was lower than in tested healthy infants (MFI median 22.6 vs. 33.2).

**Figure 8:**
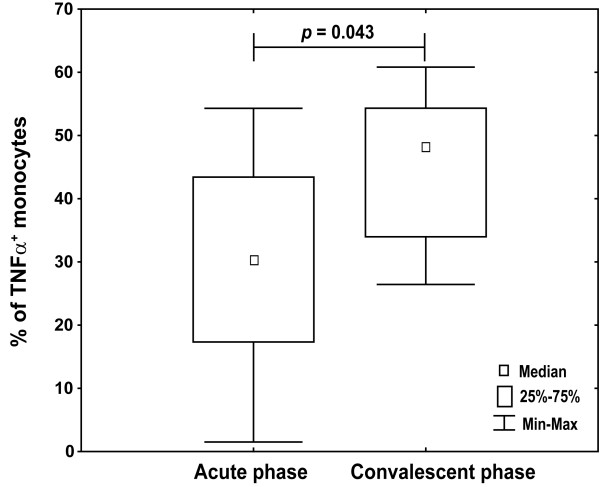
**Increased percentages of monocytes producing TNF-alpha in the convalescent phase**. Fresh PBMC from RSV-infected infants (n = 10) in acute phase and convalescent phase 4-6 weeks after first sampling were stimulated with 5 μg/mL CL075 for 6 h in the presence of brefeldin A. Intracellular TNF-α was stained with anti-TNF-α APC-conjugated antibody in CD68-positive monocytes upon permeabilization. Non-parametric Kruskal-Wallis ANOVA test was acquired to confirm statistical significance and Mann-Whitney U-test was applied for intergroup analysis.

In the convalescent phase more monocytes produced TNF-α than in acute infection which was in concordance with observed difference in the TLR8 expression (Figure 8). However, lower percentages of monocytes from infected infants in the convalescent phase than from healthy controls produced TNF-α (median 49.1 vs. 77.8%).

**Figure 9:**
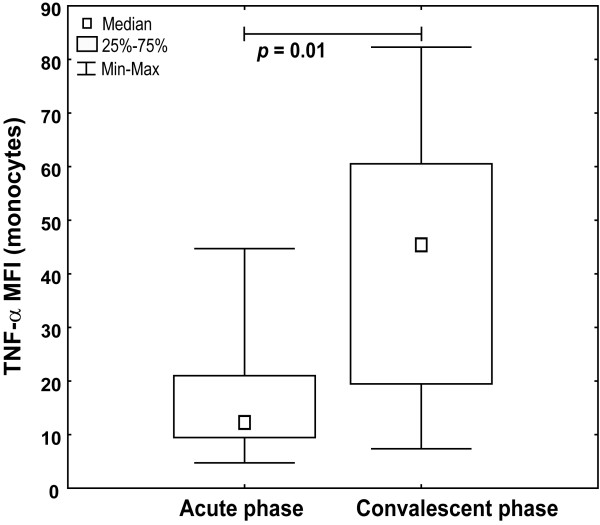
**Increased TNF-alpha levels in monocytes in the convalescent phase**. Fresh PBMC from RSV-infected infants (n = 10) in acute phase and convalescent phase 4-6 weeks after first sampling were stimulated with 5 μg/mL CL075 for 6 h in the presence of brefeldin A. Intracellular TNF-α was stained with anti-TNF-α APC-conjugated antibody in CD68-positive monocytes upon permeabilization. Non-parametric Kruskal-Wallis ANOVA test was acquired to confirm statistical significance and Mann-Whitney U-test was applied for intergroup analysis.

TNF-α expression levels in the convalescent phase followed a positive trend observed for monocyte percentages, reaching levels of healthy infants (MFI median 45.4 vs. 52.3), although still lower than healthy adults (Figure 9).

### Decreased TLR8 expression and TNF-α synthesis correlate with disease severity

We also determined whether the severity of illness correlated with observed variations in the TLR8 expression in monocytes of infected infants. Severity of illness was assessed by clinical parameters such as wheezing duration, minimal oxygen saturation, oxygen supplementation, heart rate, maximal respiratory frequency (referring to respiratory pathway obstruction) and duration of hospitalization. The levels of TLR8 in monocytes inversely correlated to maximal respiratory frequency (Table 2), emphasizing possible correlation with lung obstruction. Moreover, analysis of the TLR8-mediated cytokine synthesis upon specific stimulation revealed positive correlation between TNF-α productions with minimal oxygen saturation in hospitalized RSV-infected infants (Table 2).

**Table 2:** Correlations of clinical data and experimental findings.

Clinical findings	% TLR8^+ ^monocytes (n = 15)	TLR8 MFI in monocytes (n = 15)	% TNF-α^+ ^monocytes (n = 10)	TNF-α MFI in monocytes (n = 10)
Age (months)	-0.40	-0.41	0.01	-0.27

WBC count (x10^9^/L)	0.23	0.34	-0.48	-0.09

Wheezing duration (days)	0.02	0.32	-0.23	-0.55

MOS (%)	-0.01	-0.05	**0.73**	**0.66**

Oxygen supplementation (days)	0.22	0.10	0.16	0.31

Heart rate (/min.)	-0.11	-0.45	-0.05	0.19

MRR (/min.)	-0.05	**-0.59**	0.37	0.26

Hospital stay (days)	-0.21	-0.16	0.18	0.55

Overall statistical correlations of clinical data from RSV infected infants and experimental findings were assessed by Spearman rank correlation test. Numbers represent Spearman rank correlation coefficients and those with significant p-values (≤ 0.05) are in bold.

WBC - white blood cells, MOS - minimal oxygen saturation measured by percutaneous oxymetry breathing ambient air, MRR - maximal respiratory rate (frequency) during hospitalization period

## Discussion

RSV primarily infects respiratory epithelium where it can be detected by different pattern recognition receptors [10,12,31]. In lungs, resident airway macrophages and DC also enrolled the virus and initiate early innate immune responses responsible for the virus replication control [32,33]. In early infection phase, infiltrated monocytes differentiate into macrophages, becoming the most abundant mononuclear cells in lungs [34]. The importance of macrophages in RSV infection was confirmed by their depletion that increased RSV replication [33] and augmented pathological response [35]. Therefore, monocytes entering infected lungs differentiate into macrophages or myeloid DC might participate in RSV control, sensing the virus via different receptors including the TLR-family. Human monocytes/macrophages and myeloid dendritic cells express TLR8 and could sense RNA viruses [36]. In our study we showed lower percentages of peripheral blood monocytes expressed TLR8 in RSV-infected infants compared to other groups (Figure 3). TLR8 levels were significantly decreased during acute RSV infection compared to healthy infants (Figure 4), indicating that deficient virus recognition by monocytes/macrophage could compromise efficient anti-RSV immune response and development of more severe disease. Synthetic TLR8 ligands can induce Th1 immune responses regardless of whether monocytes/macrophages or monocyte-derived DC were used [19,37-39]. Therefore, decreased TLR8 expression in monocytes from RSV-infected infants might explain lower Th1-polarizing cytokine production i.e. TNF-α, IL-12p70 and IFN-γ observed during acute RSV-infection [22,40-42]. In our study, healthy infants, compared to adults, had lower percentages of TLR8-positive monocytes but higher TLR8 expression levels that compensated for the total amount of intracellular TLR8 (Figure 4).

The functional relevance of decreased TLR8 expression in monocytes from infected infants was analyzed measuring intracellular TNF-α synthesis upon TLR8 stimulation. As expected, significantly lower percentages of monocytes from RSV-infected infants produced TNF-α compared to healthy adults and infants (Figure 5). Although Levy at all [25,43] shoved that *in vitro *TLR8 stimulation of newborn blood samples induce comparable TNF-α levels to adult blood measured by ELISA, we tested amount of TNF-α production on a single cell level by intracellular staining technique. We found that monocytes from healthy infants produced less TNF-α than adults (Figure 6) that coincide with findings by Kollmann et al [43]. Monocytes from RSV-infected infants produced even lower TNF-α levels compared to healthy infants implicating acquired innate immunity suppression frequently observed in acute viral infections, respectively.

To test whether a difference in TLR8 expression and specific TNF-α production is the transitory effect occurred during acute RSV infection, infants were retested in the convalescent phase (4-6 weeks after first sampling) and increase in TLR8 expression levels was observed but haven't reached levels from healthy infants (Figure 7). Percentages of TLR8-positive monocytes from RSV-infected infants in the convalescent phase didn't statistically differ to acute phase (data not shown). Moreover, specific TLR8 stimulation of monocytes from infants in acute infection induced less TNF-α than in the convalescent phase, measured as percentage of monocytes producing TNF-α (Figure 8) and TNF-α MFI (Figure 9) confirming functional relevance of the observed lower TLR8 expression in diseased infants.

Since only a fraction of all RSV-infected infants develop severe LRT disease and virtually all children become infected at least once by the age of two, it may be that decreased TLR8 expression predisposes for severe disease. In our study TLR8 levels in monocytes negatively correlated with disease severity, described as tachypnea (Table 2). In concordance, percentage of monocytes producing TNF-α during acute RSV infection positively correlated with minimal oxygen saturation (Table 2) upon TLR8 stimulation. Both findings implicate impaired early innate immune response in infants with RSV-bronchiolitis linked to lower TLR8 expression and subsequent lower TNF-α release, that has been associated with acute RSV infection [44,45]. Moreover, recently published analysis of TLR8 polymorphism linked to allergic asthma, indicate importance of TLR8 in type 1 vs. type 2 immune response balance that has been also impaired in infants with severe RSV infection [46].

Opposite to our findings, acute viral infections would increase TLR8 expression via type I IFN autocrine mechanism, respectively. Increased mRNA for TLR8 were detected in PBMC from infants with acute rotavirus infection [47,48]. Interestingly, Th2 cytokines like IL-4 and IL-13 downregulate toll-like receptors involved in anti-viral immune response [49] and could explain our findings of lower TLR8 expression, since RSV causes significant Th2 cytokine production [22,42] versus Th1 cytokine response predominantly found in infants infected with rotavirus [48]. Whether the observed decreased TLR8 expression is RSV-specific, infants with other viral infections should be investigated.

## Conclusions

Lower TLR8 expression in monocytes during acute RSV infection might have a dampening effect on the early anti-viral cytokine production, upon RSV recognition, necessary to control viral infection and leading to severe LRT disease in infected infants. Depressed TLR8 expression in the convalescent phase might contribute to the subsequent RSV reinfections, frequently observed in infants.

## Competing interests

The authors declare that they have no competing interests.

## Authors' contributions

KB contributed to conception and study design, acquired in vitro experiments and drafted manuscript. VV carried out immunoassays and flow cytometric data analysis. NA and JC-B collected clinical samples and performed clinical data analysis. AG revisited critically for important intellectual content. GM-G carried out RSV diagnostic assay. SR made contributions to concept and study design.

All authors read and approved the final manuscript.

## Supplementary Material

Additional file 1 **Figure S1: Representative figure of unstimulated and CL075-stimulated monocytes stained with CD14 antibody**. Fresh PBMC from healthy adult were stimulated with 5 μg/mL CL075 or cultured in media only for 6 h, in the presence of brefeldin A. Surface CD14 staining was performed. Staining with isotype matched control antibody is also included. Click here for file

Additional file 2 **Figure S2: Representative figure of unstimulated and CL075-stimulated monocytes stained with CD68 antibody**. Fresh PBMC from healthy adult were stimulated with 5 μg/mL CL075 or cultured in media only for 6 h, in the presence of brefeldin A. Intracellular CD68 staining was performed. Staining with isotype matched control antibody is also included. Click here for file

Additional file 3 **Figure S3: Determination of optimal CL075 concentration for in *vitro culture***. Optimization was performed using fresh adult PBMC (n = 3) and increasing CL075 concentrations (2.5-10 μg/mL). Production of TNF-α in monocytes, as well lymphocyte and monocyte cell count ratio were acquired after 6 hours of culture. Columns represent ratio between monocyte and lymphocyte counts, expressed as monocyte percentages where 100% represent ratio in PBMC cultured in media only. Dots within columns represent TNF-α producing monocytes expressed as percentage. Values are median values of three tested adults. Click here for file
