# Supplementary figures and images for: Decreased Toll-like receptor 8 expression and lower TNF-alpha synthesis in infants with acute RSV infection
Source: Respir Res. 2010 Oct 14;11(1):143. doi: 10.1186/1465-9921-11-143 (PMC2974671; doi:10.1186/1465-9921-11-143)

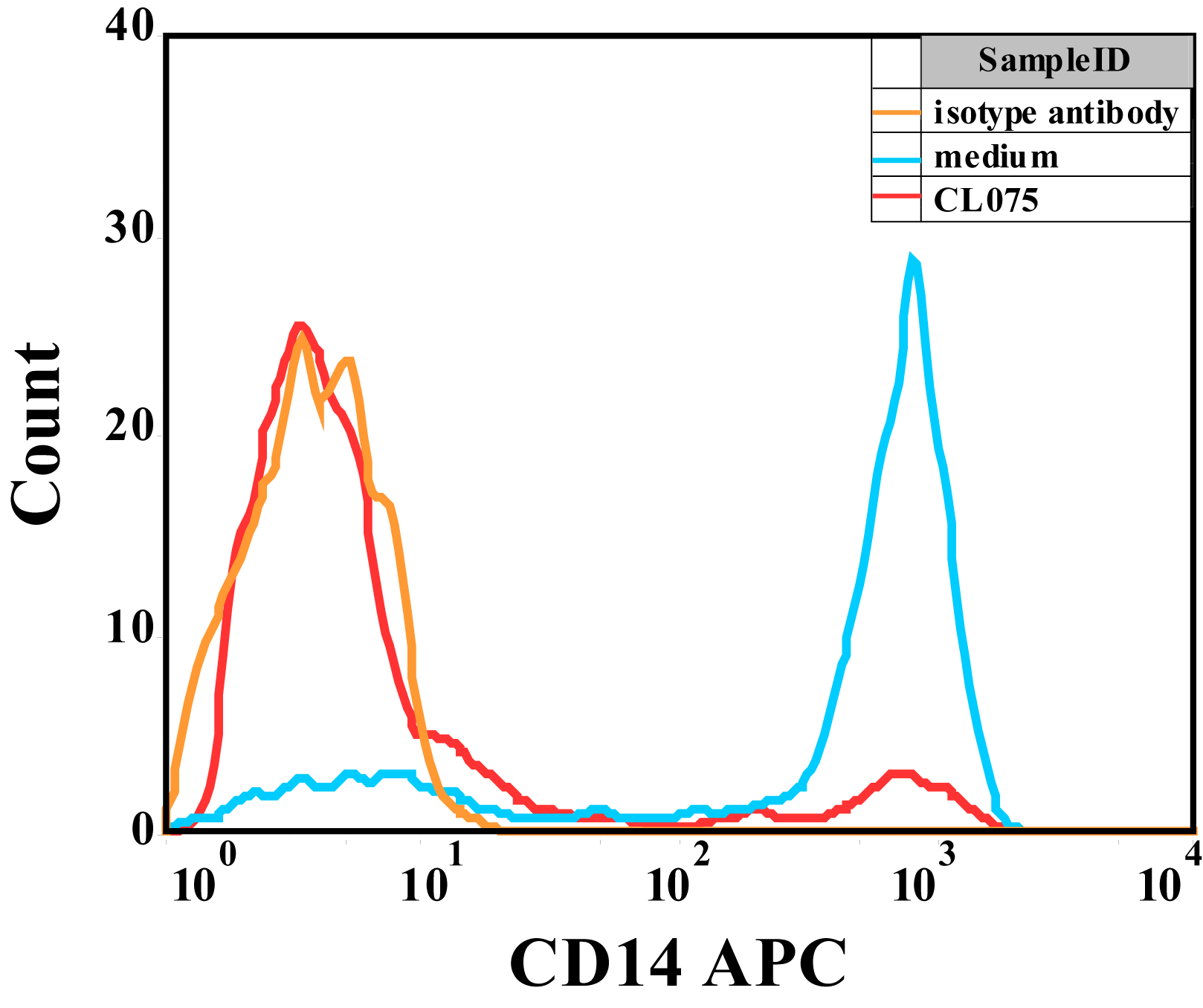

Supplement: Additional file 1 — Figure S1: Representative figure of unstimulated and CL075-stimulated monocytes stained with CD14 antibody. Fresh PBMC from healthy adult were stimulated with 5 μg/mL CL075 or cultured in media only for 6 h, in the presence of brefeldin A. Surface CD14 staining was performed. Staining with isotype matched control antibody is also included. [file 1465-9921-11-143-S1.tiff]

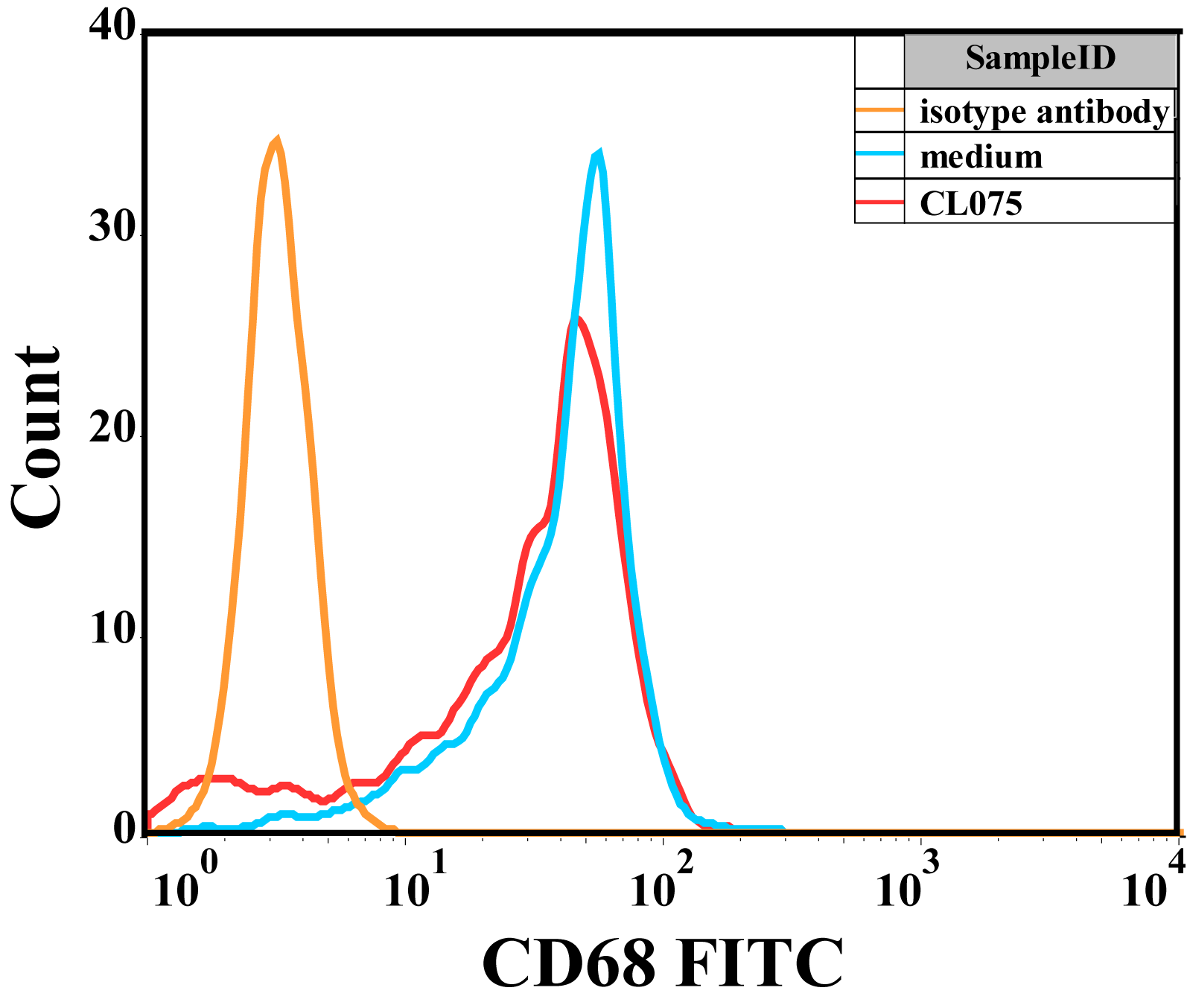

Supplement: Additional file 2 — Figure S2: Representative figure of unstimulated and CL075-stimulated monocytes stained with CD68 antibody. Fresh PBMC from healthy adult were stimulated with 5 μg/mL CL075 or cultured in media only for 6 h, in the presence of brefeldin A. Intracellular CD68 staining was performed. Staining with isotype matched control antibody is also included. [file 1465-9921-11-143-S2.tiff]

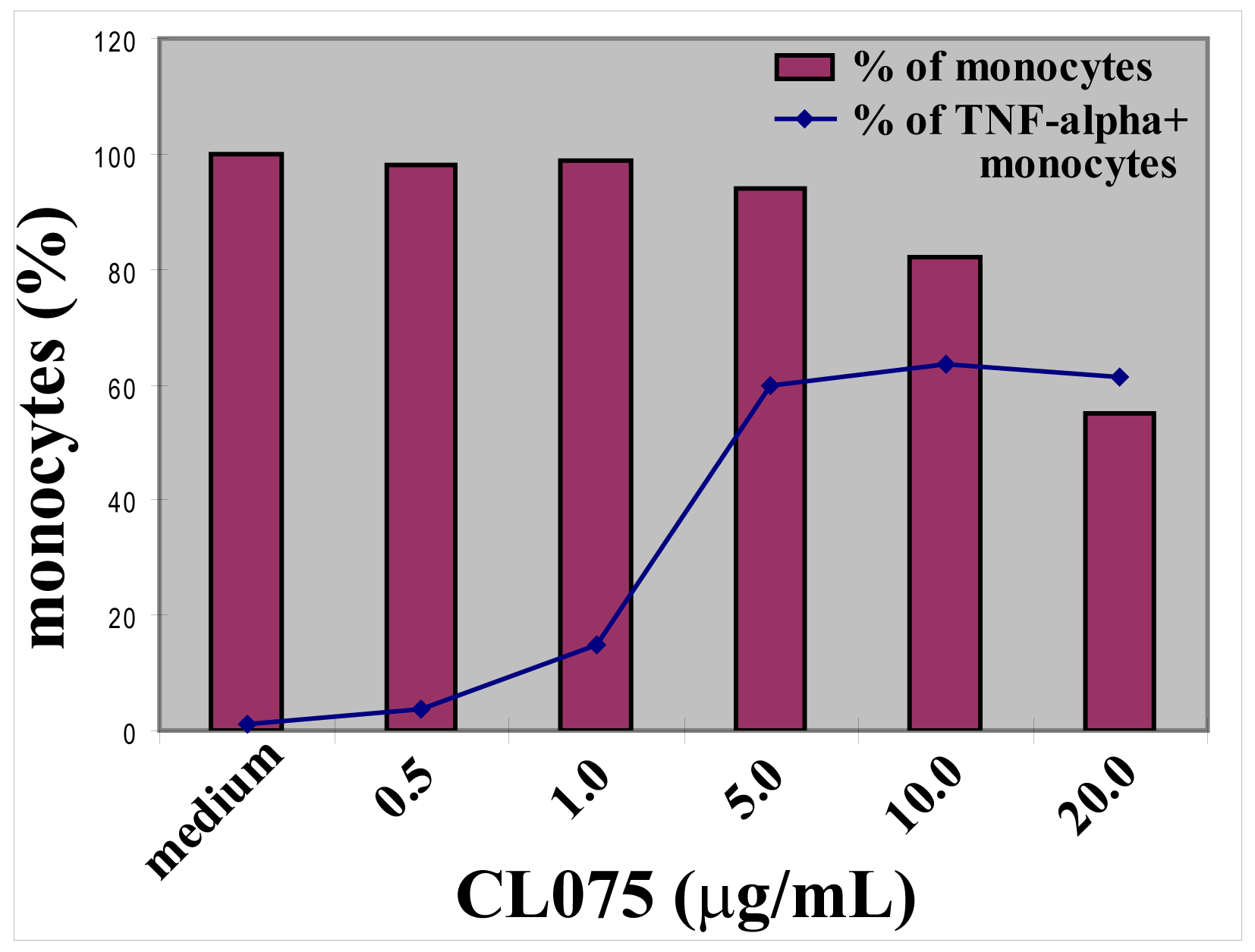

Supplement: Additional file 3 — Figure S3: Determination of optimal CL075 concentration for in vitro culture. Optimization was performed using fresh adult PBMC (n = 3) and increasing CL075 concentrations (2.5-10 μg/mL). Production of TNF-α in monocytes, as well lymphocyte and monocyte cell count ratio were acquired after 6 hours of culture. Columns represent ratio between monocyte and lymphocyte counts, expressed as monocyte percentages where 100% represent ratio in PBMC cultured in media only. Dots within columns represent TNF-α producing monocytes expressed as percentage. Values are median values of three tested adults. [file 1465-9921-11-143-S3.tiff]
